# Enteral Nutrition in the Management of Pediatric and Adult Crohn’s Disease

**DOI:** 10.3390/nu10050537

**Published:** 2018-04-26

**Authors:** Tawnya Hansen, Donald R. Duerksen

**Affiliations:** Max Rady College of Medicine, Department of Medicine, Section of Gastroenterology, University of Manitoba, St. Boniface Hospital, Winnipeg, MB R2H 2A6, Canada; Tawnya.Hansen@gmail.com

**Keywords:** enteral nutrition, Crohn’s disease, pediatric, microbiome, fistula, Crohn’s disease therapy

## Abstract

Genetic and environmental factors are thought to profoundly influence the pathophysiology of Crohn’s disease (CD). Changes in dietary and hygiene patterns affect the interactions between the immune system and environment. The gut microbiome is responsible for mediating host immune response with significant dysbiosis observed in individuals with CD. Diet therapy using exclusive enteral nutrition (EEN) has been studied as primary therapy for the management of CD. EEN may cultivate the presence of beneficial microbiota, improve bile acid metabolism, and decrease the number of dietary microparticles possibly influencing disease and immune activity. In this review, we will address the current evidence on EEN in the management of adult and pediatric CD. In adults, EEN appears to be moderately beneficial for the induction of remission of CD; however, its use is understudied and underutilized. Stronger evidence is in place to support the use of EEN in pediatric CD with the added benefit of nutrition support and steroid-sparing therapy during the growth phase. Overall, EEN is an established therapy in inducing CD remission in the pediatric population while its role as primary therapy of adult Crohn’s disease remains to be defined.

## 1. Introduction

Crohn’s disease (CD) is an incurable chronic inflammatory condition of the gastrointestinal tract. The goals of therapy are to attain histologic and clinical remission through the complementary use of pharmacologic, nutritional, and surgical therapies [1]. In the 1970s, Voitk et al. were the first to report on the value of enteral nutrition (EN) in the management of active inflammatory bowel disease (IBD). After providing their patients with EN for 3 weeks, they noted improved inflammatory indices and nutritional states leading them to postulate whether EN could be a primary therapy [2]. Subsequently, studies have evaluated the role of EN in the induction and maintenance of remission of adult and pediatric CD.

As a primary therapy, EN provides the complete daily nutritional requirements through liquid formulations delivered orally or through nasogastric or gastrostomy tubes over a period of 6–8 weeks [3]. Enteral nutrition has shown efficacy in controlling disease activity, maintaining clinical remission, and addressing malnutrition in pediatric and adult CD patients with active disease [4,5]. However, the mechanism of action is incompletely understood. Proposed pathways include altering microbiome diversity, decreasing dietary microparticles and modulating bile acid metabolism [6,7,8]. Additionally, the optimal formula to achieve maximal clinical response is also unclear [9,10]. Wide ranges of formulations are commercially available, and decisions regarding formula choice are influenced by peripheral factors such as patient co-morbidities and volume status [6]. In this review, we will provide a synopsis of EN formulations, discuss the possible mechanisms of exclusive enteral nutrition (EEN) action and evaluate the current evidence on EN in the management of CD in pediatric and adult patients. Information regarding enteral nutrition in the management of malnutrition and micronutrient deficiencies in CD will not be included in this review.

## 2. Enteral Nutrition Formulas

EN formulations can be classified as whole protein (polymeric), modified protein (elemental/semi-elemental) and disease specific [6]. Polymeric formulas are approximately 45–60% carbohydrate, 15–20% protein, and 30–40% fat. Sources of carbohydrates, proteins, and fats include corn syrup, soybean protein and canola, soybean, sunflower, and corn oil as well as MCT, respectively. These nutrients are provided in proportions meant to meet the daily macronutrient intake of healthy individuals [6]. Since these macronutrients are intact, polymeric diets are best suited for individuals with normal digestive and absorptive capabilities. They are also the most palatable when consumed orally. In contrast, modified protein formulas contain digested macronutrients. Semi-elemental formulations include oligopeptides, dipeptides or tripeptides, and medium chain triglycerides [11]. Elemental formulas contain wholly hydrolyzed macronutrients such as amino acids and simple sugars with low-fat content [6,11]. Modified protein formulas are best suited for individuals with gastrointestinal dysfunction and malabsorption but are generally used second line after an individual has failed a polymeric formulation because of cost, availability, and palatability [6]. In the management of CD, meta-analysis data suggests that there is no statistically significant difference between EN formulations in inducing CD remission [12]. Large randomized controlled trials comparing various formulations are needed to determine superiority.

## 3. Mechanism of Action

No causative dietary component has been found to reliably trigger the development of CD [13]. However, evidence does suggest that dietary elements may support or discourage pro-inflammatory states. Modulation of the microbiome to favor an anti-inflammatory state is a leading hypothesis for the mechanism of action of EEN [14]. The intestinal microbiome in CD is characterized by decreased diversity with an overrepresentation of pro-inflammatory organisms such as invasive Enterobacteriaceae [15]. This dysbiosis is thought to support pathologic cytokine production in comparison to commensal organisms that encourage CD4 cells to produce cytokines that support immune homeostasis [16,17,18]. Furthermore, bacterial metabolism generates short chain fatty acids that have a crucial role in maintaining mucosal defense [7,19]. Compositional changes in the microbiome can occur rapidly after dietary modification and have an impact on gastrointestinal health [20]. For example, diets high in animal fats have been shown to generate increased levels of intestinal deoxycholic acid via bacterial metabolism, a secondary bile acid associated with liver cancer [20,21]. Along the same line, EEN has been found to alter the microbiome resulting in improved CD activity [22]. Paradoxically, studies have demonstrated an unexpected decrease in beneficial bacterial taxa during EEN [22,23]. Gerasimidis et al. collected stool samples from a pediatric population of 15 CD and 21 healthy controls and paradoxically found decreased populations of Faecalibacterium prausnitzii and Bacteroides during the period of clinical improvement while on EEN. Interestingly, microbial diversity increased after participants resumed their normal diets [23]. Beyond modulating the intestinal microbiome, EEN is also thought to have a direct effect on enterocytes [24]. In an in vitro model, de Jong et al. showed that enterocytes exposed to inflammatory stimuli responded less vigorously if they had been incubated with polymeric formula. The authors hypothesized that polymeric formula might act directly on enterocytes to alter intracellular signaling thereby lowering the production of pro-inflammatory interleukin 8 (IL-8) [24]. Further research in this area has suggested that glutamine and arginine, both found in polymeric formulas, may block phosphorylation within the nuclear factor (NF)-kB pathway resulting in decreased IL-8 production [14]. Similar findings have been reported with colonocytes incubated with elemental formula [25]. In clinical studies, investigators have demonstrated a decrease in cytokine production and evidence of mucosal healing in response to enteral nutrition [26,27].

The influence of dietary microparticles is another mechanism of action hypothesis albeit controversial [8]. Microparticles are tiny inorganic compounds found in medications and processed foods. Common microparticles are titanium dioxide and aluminosilicates that serve as whitening and anti-caking agents, respectively. After consumption, these molecules accumulate in the macrophages residing within Peyer’s patches. As these macrophages come into contact with and mount inflammatory responses to pathogen associated molecular patterns, these microparticles potentiate cytokine response and increase oxidative damage possibly contributing to the development of CD [8]. Elemental diets are thought to be free of microparticles and thus may have a beneficial effect on CD activity. Lomer and colleagues evaluated the effect of low dietary microparticles on CD in 2 separate studies [28,29]. Their pilot study reported significant improvements in the Crohn’s Disease Activity Index (CDAI) in individuals with ileal CD on corticosteroids after 4 months of diet therapy [28]. However, these findings were not reproduced in a more extensive study of 82 individuals followed out to a year [29].

## 4. Exclusive Enteral Nutrition in the Induction of Remission in Pediatric Crohn’s Disease

The incidence of pediatric Crohn’s disease (pCD) varies by region and in North America is estimated to be approximately 4.56 per 100,000 to 11.4 per 100,000 [30,31]. In comparison to adult-onset CD, pCD generally presents with a more aggressive and extensive disease phenotype. Aside from luminal or perianal inflammation, children can also present with weight loss, impaired bone growth, and pubertal delay. As such, EEN is especially favored in this population for the management of mild-moderate pCD over corticosteroids that can have detrimental effects on development [4].

Meta-analysis data suggests that EEN is equal in efficacy to corticosteroids in inducing clinical remission [32,33]. When subgroups have been examined, a trend towards higher response rates can be observed among first presentation CD cases in comparison to relapsed cases [34]. Second course EEN has been identified as a risk factor for unsustained remission at 3 months in a retrospective analysis of 52 pCD cases [35]. This study also stratified genetic profiles and found differences in outcome. Individuals with the NOD2 R702W genotype had a 92% relapse rate compared to 60% for the wild type (*p* < 0.01) [35]. Several studies have evaluated whether disease phenotype can predict poor EEN response; however, results have been conflicting [12,36,37,38].

Exclusive enteral nutrition has also been prospectively compared to infliximab [39]. In a single center study, 26 pCD patients were divided equally into EEN and infliximab treatment arms. Clinical response was assessed using the pediatric Crohn’s disease activity index (PCDAI) after 8 weeks of therapy. Statistically significant differences in PCDAI scores were noted in both groups when comparing baseline to 8-week scores however there was no significant difference in remission rates between groups. Mucosal healing (MH) was assessed in 14 patients (7 from each treatment arm) at 8 weeks, and 5 individuals in the EEN group achieved MH compared to 6 in the infliximab group. No children in the EEN treatment arm experienced any adverse effects. However, adverse events were noted in the infliximab arm and included 2 infusion reactions requiring methylprednisolone administration, 1 seizure during the 3rd dose infusion and 1 child with recurrent upper respiratory infections [39]. EEN appears to be an efficacious treatment for inducing remission in pCD and is at least as efficacious as corticosteroid therapy. More research is needed to determine how EEN compares to infliximab. Therapeutically, significant benefits of EEN include the excellent side effect profile, positive effects on growth and high rate of mucosal healing [40]. These benefits are offset by factors such as cost, palatability, and compliance.

## 5. Supplemental Enteral Nutrition and the Maintenance of Remission in Pediatric Crohn’s Disease

EEN is generally used for a 6–12-week period during which longer-term pharmacologic therapies such as azathioprine may or may not be initiated [4] Upon completion of the induction period, a regular diet is slowly introduced. Less often, EN has been used post-induction in intermittent cyclic administration or as a supplement. There appear to be benefits with this limited use during the maintenance phase in the form of longer remission periods [41] and improved linear growth [36]. However, the role of supplemental nutrition in the maintenance of remission has not been well studied in the pediatric population.

After EEN induction, long-term remission rates have been reported to be similar to or slightly better when compared to corticosteroid induction with the added benefits of improved growth rates, normalization of bone metabolism and increased steroid free survival [42,43,44,45,46,47,48,49]. One of the first studies to prospectively report long-term outcomes in children treated with EEN was conducted by Grogan and colleagues [50]. Their double blind randomized controlled trial compared remission rates among children receiving elemental formula versus polymeric formula. Children were followed for 24 months with 15 receiving elemental formula and 19 receiving polymeric formula. In both groups, approximately one-third of participants did not experience a relapse at 2 years. Additionally, the mean number of days until relapse was not significantly different (elemental formula: 183 days; polymeric formula: 162 days) [50]. More recently, the GROWTH CD study, prospectively followed 147 children with mild to moderate CD for 2 years to evaluate steroid-free remission and growth rates [42]. At 78 weeks, children treated with EEN had a trend towards higher Z scores when compared with children treated with CS. Remission rates were significantly better in the EEN group (63% vs. 47%; *p* = 0.036) however relapse rates at 78 weeks were similar between both groups. Lafferty et al. also reported similar findings in a case-matched retrospective chart review [43]. Children in the EEN arm attained higher remission rates when compared to the CS (86% vs. 54%, *p* = 0.02) with continuous dietary counseling being integral to success. At a median follow up of 2.04 years, height Z scores were significantly improved. Positive outcomes were found most commonly when EEN was used as the initial treatment and children >10 years old [43].

Inducing remission with EEN may decrease the longer-term need for steroid and anti-TNF therapies. These observations were reported in an observational study of 111 propensity-score matched cases of pCD [45]. Of the patients initially treated with EEN, 39.6% and 94.7% were steroid and anti-TNF naïve at 4 years, respectively. This effect, however, did not significantly alter the rates of CD-related surgeries when compared to children induced with steroids [51]. Individuals who will go on to have relapse free survival may be identified at post-EEN endoscopy. In a prospective cohort study of 54 newly diagnosed pCD patients treated with a median 8.57 weeks of enteral nutrition and immunomodulation, the most reliable predictor of sustained remission was post-EEN mucosal healing [52]. In that population, 50% of children with MH after EEN induction had a subsequent relapse whereas 94% of children with endoscopic activity had relapsed at 3 years (*p* = 0.005) [52]. Thus, patients with active endoscopic disease after induction are likely at high risk of relapsing and may need to be observed more closely for therapy escalation.

## 6. Exclusive Enteral Nutrition in the Induction of Remission of Adult Crohn’s Disease

Corticosteroids remain the first line treatment to obtain rapid clinical remission in adult CD. Studies suggest that steroids are likely superior to EEN in adult patients, but reasons for this are unknown [3,12]. A Cochrane meta-analysis published in 2007 evaluated 6 trials (including 1 pediatric trial) and obtained a pooled odds ratio of 0.33 favoring steroid therapy over EEN (95% CI 0.21–0.53) [12]. These findings were supported by a more recent systematic review as well [53].

EEN has been studied in complicated Crohn’s disease with positive results. A prospective, observational study followed 41 CD patients with fistulas, strictures, and abscesses [54]. All patients were given EEN while individuals with abscesses also received antibiotics with or without the addition of drainage. After 12 weeks of therapy, 80.5% of patients achieved clinical remission, and 75% of patients with enterocutaneous fistulas experienced fistula closure [54]. Marked improvement in inflammatory strictures was also observed another prospective cohort study where 81.4% of patients achieved clinical remission with a 331% reduction in luminal the cross-sectional area found on imaging [55]. In comparison, fistula closure rate with infliximab has been reported at 68% [56]. It is hard to make direct comparisons and conclusions are limited because of small numbers, but the results of these EEN studies are promising. 

## 7. Supplemental Enteral Nutrition and Maintenance of Remission in Adult Crohn’s Disease

Enteral nutrition can be used as maintenance therapy for CD. Takagi et al. compared supplemental enteral nutrition (*n* = 26) to an unrestricted diet (*n* = 25) as a maintenance strategy for individuals who underwent induction of remission with enteral nutrition, steroids and/or infliximab [57]. At a mean follow up of 11.9 months, 64.0% of individuals receiving the supplemental elemental diet were in remission compared to 34.6% of persons on a free diet (multivariate hazard ratio 0.4, 95% CI: 0.16–0.98). A second study evaluating the efficacy of supplemental polymeric to elemental formula for maintenance did not find any significant differences [58]. These 2 randomized controlled trials were analyzed in a 2007 Cochrane review, but data analysis was limited by small sample sizes [5]. Similarly, an updated systematic review and meta-analysis also suggested that supplemental enteral nutrition is likely beneficial but once again were limited by the quality of the data [59,60]. Supplemental enteral nutrition likely represents a more flexible approach to nutritional therapy that is more acceptable for quality of life and could be more readily implemented [61].

More robust data has been published on patients on infliximab and supplemented with EN. A recent meta-analysis included 4 studies and evaluated the benefit of IFX and enteral nutrition with IFX monotherapy for the maintenance of clinical remission [62]. There were 157 patients on IFX and EN and 185 patients on IFX monotherapy. Patients were supplemented with at least 600 kcal/day with elemental or polymeric nutritional feeds. The rate of clinical remission in patients receiving EN with IFX was 69.4% versus 45.4% in those with IFX monotherapy. They determined that the number needed to treat with EN to maintain clinical remission was 4 patients. No heterogeneity between studies was noted. Clinical remission rates at 1 year were still significantly higher in the EN group compared to the IFX group (74.5% vs. 49.2%, OR 2.93; 95% CI: 1.66–5.17, *p* < 0.01) [62]. This benefit also appears to extend to preventing CD recurrence post resection up to 5 years post operation [63].

EEN has also been studied in the pre-operative setting with positive results. Heerasing and colleagues performed a retrospective review of patients with stricturing or penetrating complications of CD scheduled for surgery [64]. Among these patients, 51 were identified who received EEN before surgery and they were matched to controls. After EEN, 25% (13/51) of patients no longer required surgery. Operative outcomes were also improved with EEN treated patients experiencing fewer surgical complications (8% vs. 32%, *p* < 0.001). No differences were seen in recurrence rates up to one-year post surgery [64].

## 8. Implementation of Exclusive Enteral Nutrition

The initiation of EEN can be a daunting task for the patient and caregiver. A multidisciplinary team is needed to offer technical, medical, and nutritional support throughout the process. Prior to starting EEN, a comprehensive nutritional assessment by a dietician is required [65]. Malnutrition and individual caloric needs should be identified and continuously re-evaluated. Dieticians are also influential in providing education to patients and introducing them to various products that are available to provide nutrition. The dietician’s role is integral to creating a dynamic management plan that ensures the patient receives appropriate nutrition care, which in the pediatric age group includes attention to the growth trajectory [65]. Regarding enteral formula access, social work expertise may be required to determine optimal ways of obtaining formulae in the context of financial constraints. For individuals that choose to use nasogastric feeding tubes, nursing education is required to demonstrate proper insertion technique. Patients and caregivers also need to be instructed on techniques of administration, proper oral care, and maintenance of skin barrier. Furthermore, the treating physician must communicate expectations and identify any obstacles that may prevent the successful implementation of the treatment plan. Close follow up with the treating physician, nurse and dietician are required to maximize the chance of response.

## 9. Conclusions

Enteral Nutrition is a valuable therapy in the management of CD. Enteral nutrition likely influences the intestinal bacterial milieu, shifting the environment away from a pro-inflammatory state. EEN is an established primary therapy for mild-moderate pediatric CD with uptake dependent on regional practices and patient preferences. Its primary advantages include induction of remission while limiting steroid exposure during crucial growth stages and providing the nutritional support to meet developmental milestones. In the adult population, the utilization of EN is low with factors such as patient preference, provider comfort and increasing therapeutic options with biologic therapy influencing its use. Despite this, there remains a moderate amount of evidence supporting its use. The value of EEN should be discussed with patients and especially considered in individuals desiring a non-pharmacologic approach to managing CD and those with complicated CD associated with malnutrition. Further studies in adults are needed to evaluate the true potential of EEN and determine a place for its use in the management algorithm for broader uptake.
